# Rapid and direct synthesis of complex perovskite oxides through a highly energetic planetary milling

**DOI:** 10.1038/srep46241

**Published:** 2017-04-07

**Authors:** Gyoung-Ja Lee, Eun-Kwang Park, Sun-A Yang, Jin-Ju Park, Sang-Don Bu, Min-Ku Lee

**Affiliations:** 1 Nuclear Materials Development Division, Korea Atomic Energy Research Institute, 989-111 Daedeok-daero, Yuseong-gu, Daejeon 34057, Republic of Korea; 2 Department of Physics and Research Institute of Physics and Chemistry, Chonbuk National University, Jeonju 54896, Republic of Korea

## Abstract

The search for a new and facile synthetic route that is simple, economical and environmentally safe is one of the most challenging issues related to the synthesis of functional complex oxides. Herein, we report the expeditious synthesis of single-phase perovskite oxides by a high-rate mechanochemical reaction, which is generally difficult through conventional milling methods. With the help of a highly energetic planetary ball mill, lead-free piezoelectric perovskite oxides of (Bi, Na)TiO_3_, (K, Na)NbO_3_ and their modified complex compositions were directly synthesized with low contamination. The reaction time necessary to fully convert the micron-sized reactant powder mixture into a single-phase perovskite structure was markedly short at only 30–40 min regardless of the chemical composition. The cumulative kinetic energy required to overtake the activation period necessary for predominant formation of perovskite products was ca. 387 kJ/g for (Bi, Na)TiO_3_ and ca. 580 kJ/g for (K, Na)NbO_3_. The mechanochemically derived powders, when sintered, showed piezoelectric performance capabilities comparable to those of powders obtained by conventional solid-state reaction processes. The observed mechanochemical synthetic route may lead to the realization of a rapid, one-step preparation method by which to create other promising functional oxides without time-consuming homogenization and high-temperature calcination powder procedures.

Complex perovskite oxides containing suitable chemical elements are highly versatile and present a broad range of useful functionalities with unique physico-chemical properties. Traditionally, the majority of these materials have been produced by a solid-state reaction process, in which several intricate processing steps, including a long period of mixing or grinding of the reactant powders, multiple and prolonged calcination steps at high temperatures, and intermediate wet milling processes, are essential to create the proper chemical homogeneity and the desired structures^1^,^2^.

Recently, the field of mechanochemistry has seen a considerable upsurge of interest, mainly driven by the quest for a solvent-free, direct reaction and the low-temperature synthesis of various functional oxides^3^. The mechanochemical synthetic route is simple to implement, sustainable, and highly energy-efficient when typically carried out using a milling technique. The greatest potential lies in the possibility of promoting solid-state chemical reactions by which any reactant changes into any product at room temperature, as this is possible because the reaction is triggered by means of mechanical stress instead of heat to overcome all thermodynamic reaction barriers^4^,^5^,^6^. For the past two decades, intense efforts have been devoted to preparing various functional oxides, including PbTiO_3_^7^, PbZrO_3_^8^, Pb(Zr, Ti)O_3_^9^,^10^,^11^, (Pb, La)(Zr, Ti)O_3_^12^,^13^, Pb(Mg, Nb)O_3_^14^,^15^,^16^,^17^, Pb(Mg, Nb)O_3_-PbTiO_3_^18^,^19^, BaTiO_3_^20^,^21^, NaNbO_3_^22^,^23^, KNbO_3_^24^, (K, Na)NbO_3_^22^,^25^, and BiFeO_3_^26^, mechanochemically using different types of a mill. Among them, a planetary ball mill has been the most popular type of mill adopted in mechanochemical studies of oxides, as is characterized by higher milling energy and thus more efficient in reducing the particle size of initially larger particles^27^,^28^, compared to vibratory and attrition mills.

Nevertheless, it has been difficult to stabilize a single-phase perovskite in a direct and rapid manner from the initial reactant powder mixture usually consisting of micron-sized particles, which are cheap, easily affordable and typically used in conventional solid-state synthesis. Extending the milling time (typically, from tens to hundreds of hours) has usually been ineffective when attempting fundamental chemical changes. In many cases, a nano-crystalline or amorphous structure of the reactant powders resulted, often with contamination from the milling medium. Several studies have just shown that the mechanical stress generated by ball impacts can modify the reactant powders to lower the calcination temperature and processing time^25^,^29^,^30^. As the milling time required for nano-crystallization, which is a prerequisite for desired mechanochemical reaction, significantly depends on the initial particle size^31^, Ohara *et al*. have shown that the total duration to achieve the mechanochemical transformation can be greatly reduced by using the reactant powders at the nanoscale^21^.

The lack of the rapid and direct synthesis of a perovskite product by a planetary ball mill is attributed to a kinetic limitation associated with difficulties in developing a mill capable of providing highly energetic impacts that are critical to manage the mechanochemical reaction and related yield. Theoretical modeling of kinematic equations describing the energy transfer of the ball to the powder in a planetary mill was well established by Burgio *et al*.^32^. Kinetic considerations of the mechanochemical behavior of alkali niobate oxide by correlating the milling parameters were also formulated by Rojac *et al*.^33^. Although the experimental details from different studies were in some cases incomplete, thus thwarting deeper insight into this kinetic issue, our extensive literature survey and the calculation based on Burgio’s model indicated that the existing total energy transferred to the powder or cumulative kinetic energy (as determined by the ball-impact energy, ball-impact frequency and milling time) afforded by planetary mills was typically less than ca. 35 kJ/g for 1 h of milling, depending on design of the mill, the milling medium, the milling speed, and other related factors (Table 1).

Given that the level of energy and number of impacts transferred to the reactant powders may be correlated with an intrinsic activation energy barrier of the reaction system, at a higher activation energy level of a given reaction, a more energetic milling condition is needed to cause a reaction between starting materials mechanochemically. This also helps to facilitate chemical reactions at a faster rate and minimize chemical losses and/or contamination. From this perspective, we show here that single-phase perovskite oxides can be expeditiously synthesized from micron-sized reactant powders via a mechanochemical reaction by a highly energetic ball milling process, especially with significantly higher ball-impact energy and frequency levels. Experimental results are presented for two widely studied piezoelectric oxides, (Bi_0.5_Na_0.5_)TiO_3_ (BNT) and (K_0.5_Na_0.5_)NbO_3_ (KNN), as well as their modified complex compositions, 0.76(Bi_0.5_Na_0.5_)TiO_3_-0.04(Bi_0.5_Li_0.5_)TiO_3_-0.2(Bi_0.5_K_0.5_)TiO_3_ (BNT-BLT-BKT) and 0.955 (K_0.48_Na_0.52_)NbO_3_-0.03Bi_0.5_(Na_0.7_K_0.2_Li_0.1_)ZrO_3_-0.01(Bi_0.5_Na_0.5_)TiO_3_ (KNN-BNKLZ-BNT), as the solid-state syntheses of BNT- and KNN-based oxides and their chemical modifications are currently major challenges facing research on lead-free piezoelectrics^34^. Using the mechanochemically derived powders, the sintering characteristics and bulk piezoelectric properties are demonstrated and compared to those obtained from a conventional solid-state reaction process.

## Methods

### Materials

The reactants used in this study were K_2_CO_3_ (≥99.0%, ~150 μm, Sigma-Aldrich), Na_2_CO_3_ (≥99.5%, ~10 μm, Sigma-Aldrich), Li_2_CO_3_ (99.997%, ~20 μm, Sigma-Aldrich), Nb_2_O_5_ (99.9%, ~2 μm, Sigma-Aldrich), Bi_2_O_3_ (99.9%, ~10 μm, Sigma-Aldrich), ZrO_2_ (99.0%, ~5 μm, Sigma-Aldrich), and TiO_2_ (≥99.9%, ~1 μm, Sigma-Aldrich), which are typically used in a conventional synthetic process. The carbonate powders of K_2_CO_3_, Na_2_CO_3_ and Li_2_CO_3_ were dried at 120 °C for 24 h before use owing to their hygroscopic characteristics. Also, all treatments of these powders, including weighing and drying, were carefully performed within a glove box filled with an Ar gas atmosphere. Stoichiometric Bi_2_O_3_-Na_2_CO_3_-TiO_2_ and K_2_CO_3_-Na_2_CO_3_-Nb_2_O_5_ powder mixtures were used for the syntheses of pure BNT and KNN, respectively. The general chemical reactions of the reactants for the formation of BNT and KNN are as follows:


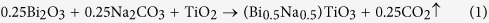



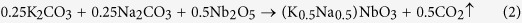


Additionally, their respective modified compositions were used as templates to assess the feasibility of preparing more complex compositions, specifically, 0.76BNT-0.04BLT-0.2BKT and 0.955KNN-0.03BNKLZ-0.015BNT, which were recently discovered to possess excellent piezoelectric properties^35^,^36^. These modified oxides were prepared using the stoichiometric Bi_2_O_3_-Na_2_CO_3_-TiO_2_-Li_2_CO_3_-K_2_CO_3_ and K_2_CO_3_-Na_2_CO_3_-Nb_2_O_5_-Bi_2_O_3_-Li_2_CO_3_-ZrO_2_-TiO_2_ powder mixtures, respectively.

### Highly energetic planetary milling and mechanochemical synthesis

Mechanochemical treatments were conducted using a planetary ball mill that was specially developed to provide the much higher milling speed required to increase both the impact energy and frequency. Several design concepts were used to maximize the rotational speed, including the anti-directional rotation mode of the disk and vial, the frictional contact between the disk and vial, the dimensions (diameter, height) of the disk and vial, the mechanical balance of the vial-embedded supporting disk at higher rotational speeds and efficient cooling with water to realize the high-speed milling operation at room temperature inside the vial (Figure S1)^37^. The vial was 5.2 cm in diameter *D*_v_ and 100 cm^3^ in volume. The distance between the rotational axes of the supporting disk and vial *R*_p_ was 5.3 cm. The ratio of the rotational angular speeds of the vial (*W*_v_) and supporting disk (*W*_p_) was 2.4 (*W*_v_ = −2.4*W*_p_). The attainable values of rotational speeds of the disk and vial were *ca*. 1100 and *ca*. 2640 rpm at a maximum, respectively. In all cases, *ca*. 5 g of a stoichiometric powder mixture was placed in a vial with balls (5, 8, 10 and 12 mm in diameter *d*_b_), both made of tungsten carbide (15.1 g/cm^3^ in density *ρ*_b_). The ball-to-powder weight ratio was kept at 36:1. Hence, the numbers of balls (*N*_b_) incorporated were 185, 45, 23 and 13 when the ball diameters were 5, 8, 10 and 12 mm, respectively. The rotational speeds of the disk and vial were set to 1000 and 2400 rpm, respectively, in the present experiments. The milling experiments were performed at different times in an argon atmosphere (*ca*. 0.3 MPa).

Four different kinetic conditions, referred to as condition numbers 1, 2, 3 and 4, were adopted for the mechanochemical treatments. The related kinetic parameters are presented in Table 1. The key parameters of the ball-impact energy Δ*E*_b_ and the ball-impact frequency *v*_t_ were controlled such that balls with a larger diameter resulted in higher Δ*E*_b_ and smaller *v*_t_ values at a constant ball-to-powder weight ratio. According to the model derived by Burgio *et al*.^32^, Δ*E*_b_ increases from 115 to 1209 mJ/hit when the ball diameter increases from 5 to 12 mm. In contrast, the corresponding value of *v*_t_ decreased greatly from 15725 to 1105 s^−1^ owing to the strong dependence of this value on the number of balls (*N*_b_) incorporated. This counterbalance yielded the cumulative kinetic energy *E*_cum_, which is directly proportional to Δ*E*_b_ and *v*_t_, between 962–1303 kJ/g for 1 h.

In Table 1, scattered kinetic data available from different studies are also presented for comparison. They clearly indicate that our milling conditions were much more energetic, as characterized by the higher values of Δ*E*_b_, *v*_t_, and *E*_cum_. Note the remarkably higher *E*_cum_ values calculated based on an identical milling time of 1 h in comparison with those from other studies. For comparison, we also prepared powders using a conventional powder process. The identical stoichiometric powder mixtures were homogenized in ethanol for 24 h using a roller mill machine operating at 70 rpm and were dried at 120 °C for 24 h. Calcination was then carried out at 850 °C for 2 h for the BNT-based powder mixtures and at 850 °C for 6 h for the KNN-based powder mixtures.

### Sintering

To investigate the sintering characteristics, the powders prepared by both methods were mixed with poly-vinyl alcohol (PVA) as a binder and axially compacted into disks 10 mm in diameter and 1 mm thick under shaping pressures of *ca*. 62 MPa for the BNT-based and *ca*. 187 MPa for the KNN-based powders. After burning off the PVA at 650 °C, traditional pressureless sintering was carried out in air. The sintering conditions were identical for the powders prepared by the two synthetic methods, but the BNT- and KNN-based oxides were sintered under different conditions, specifically, 1170 °C for 3 h and 1100 °C for 10 h, respectively, as established in earlier studies of each oxide^35^,^36^.

### Characterization

The structural phases were characterized by an X-ray diffractometer (XRD; D/Max-2500; Rigaku, Tokyo, Japan) using Cu *K*α radiation at a power of 40 kV and 15 mA and at a scan speed of 1°/min. A transmission electron microscope (TEM; JEM-2100F; JEOL, Tokyo, Japan) was also used. After milling, the degree of contamination from the milling medium was examined using an inductively coupled plasma method (ICP; Optima 8300DV; Perkin Elmer, Waltham, MA, USA). A thermogravimetric analysis (TGA; Setsys Ev 1750; SETARAM, Caluire-et-Cuire, France) was conducted to examine the mechanochemical reaction of the milled powders. Approximately 50 mg of the powder was placed in a Pt/Rh crucible and heated to 1000 °C at a rate of 10 °C/min in a flowing air atmosphere. The morphology of the sintered samples was studied using a field-emission scanning electron microscope (FE SEM; Sirion; FEI, Eindhoven, Netherlands) with an operating voltage of 20 kV equipped with an energy-dispersive X-ray spectrometer (EDXS). For piezoelectric measurements of the sintered samples, both sides of the disks were polished and painted with a silver paste, after which they were fired at 650 °C for 10 min. The samples were then poled in silicone oil at room temperature. The poling field and poling time were set to 60 kV/cm and 10 min, respectively, after optimization through pre-tests of poling over time and in an electric field. The piezoelectric coefficient (*d*_33_) was measured using a piezo-*d*_33_ meter (ZJ-6B; IACAS; Beijing, China).

## Results and Discussion

**Figure 1:**
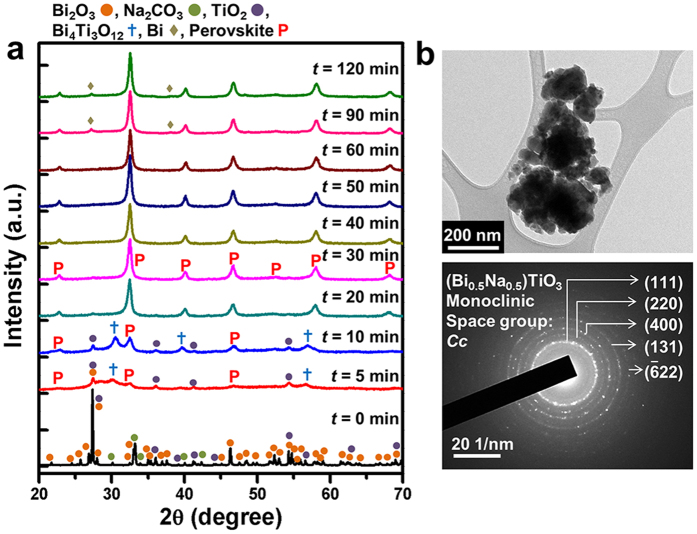
(**a**) XRD patterns of the mechanochemically treated stoichiometric Bi_2_O_3_-Na_2_CO_3_-TiO_2_ powder mixture as a function of the milling time under condition number 2 (Δ*E*_b_ = 421 mJ/hit, *v*_t_ = 3825 s^−1^). (**b**) TEM bright-field image and selected-area diffraction pattern of the powders mechanochemically treated for 40 min.

Figure 1a shows XRD patterns of the stoichiometric Bi_2_O_3_-Na_2_CO_3_-TiO_2_ powder mixture mechanochemically treated as a function of the milling time under condition number 2 (Δ*E*_b_ = 421 mJ/hit, *v*_t_ = 3825 s^−1^). With only 5–10 min of milling, most of the crystalline reactant peaks vanished due to nano-crystallization or amorphization and the characteristic diffraction peaks relevant to a perovskite structure began to appear. Meanwhile, an intermediate reaction product identified as Bi_4_Ti_3_O_12_ was detected, likely resulting from a mechanochemical reaction between Bi_2_O_3_ and TiO_2_^38^. This phase completely disappeared after 20 min. Surprisingly, a predominant perovskite structure was stabilized within 20 min, after which only a pure perovskite structure was evident, without any trace of a second phase. The diffraction peaks were indexed as BNT with monoclinic symmetry (JCPDS 01-080-4260, space group: *Cc*)^39^. After 90 min, the Bi phase was detected, indicating decomposition of the perovskite BNT. The finely crystalline characteristic of the perovski te BNT phase was estimated from the broadening of the XRD peaks; according to Scherrer’s formula, the crystallite size was *ca*. 17.0–18.5 nm. Such a structural characteristic is well supported by the TEM results shown in Fig. 1b.

Similar outcomes, such as reductions of the crystallite size, amorphization and the formation of Bi_4_Ti_3_O_12_ and perovskite BNT, were noted for the powders prepared under condition numbers 1, 3 and 4 (Figure S2). For condition number 1 (Δ*E*_b_ = 115 mJ/hit, *v*_t_ = 15725 s^−1^), however, a trace of the perovskite phase began to appear after 30 min of milling. Moreover, considerable reactant peaks such as stable TiO_2_ were observed up to 60 min, indicating an incomplete reaction during the milling process (Figure S2a). For condition numbers 3 and 4, no appreciable changes were visible compared to condition number 2, but the partly unreacted TiO_2_ remained for prolonged periods of time and the formation of the further promoted Bi phase via BNT became severe (Figure S2b and c).

**Figure 2:**
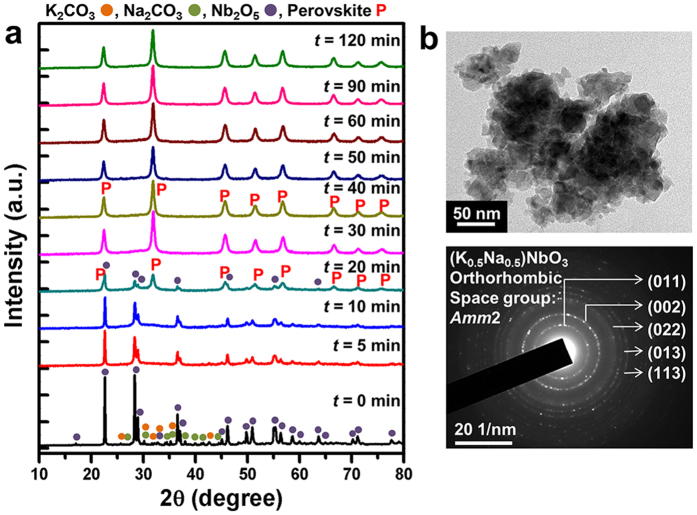
(**a**) XRD patterns of the mechanochemically treated stoichiometric K_2_CO_3_-Na_2_CO_3_-Nb_2_O_5_ powder mixtures as a function of the milling time under condition number 2 (Δ*E*_b_ = 421 mJ/hit, *v*_t_ = 3825 s^−1^). (**b**) TEM bright-field image and selected-area diffraction pattern of the powders mechanochemically treated for 40 min.

Figure 2a shows XRD patterns of the stoichiometric K_2_CO_3_-Na_2_CO_3_-Nb_2_O_5_ powder mixtures mechanochemically treated as a function of the milling time under condition number 2. The intensity of the Nb_2_O_5_ peaks rapidly decreased owing to the reduced crystallite size. The peaks belonging to the alkali metal carbonates of K_2_CO_3_ and Na_2_CO_3_ disappeared after a very short period of milling (5–10 min), accompanied by a slight increase of the background near their reflections, which indicated amorphization. The oxide-assisted amorphization of carbonates during milling is well known in *A*_2_CO_3_-Nb_2_O_5_ (*A* = K or/and Na) systems, for which the reconstruction of 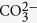 ions into a carbonato complex results in a loss of long-range periodicity of the carbonates^40^. After 30 min, the Nb_2_O_5_ peaks disappeared and the only phase detected was an orthorhombic KNN phase (JCPDS 01-071-0946, space group: *Amm*2) with a crystallite size of *ca*. 16.5–16.7 nm based on Scherrer’s formula. The formation of a nano-crystalline perovskite structure was further confirmed by TEM (Fig. 2b).

In contrast, the reactant powders, when subject to condition number 1, showed continuous peak broadening and a decreased intensity accompanied by a gradual increase in the background with an increase in the milling time. The perovskite phase began to appear after 60 min and then became dominant (Figure S3a). This was notable because the reactant powders experienced much more vigorous collisions (*v*_t_ = 15725 s^−1^) as compared to those in condition number 2 (*v*_t_ = 3825 s^−1^), clearly indicating that the rate of the mechanochemical reaction was decreased appreciably by the lower Δ*E*_b_ (=115 mJ/hit). For condition number 3, the perovskite KNN dominated after 40 min, but with the presence of unreacted Nb_2_O_5_ (Figure S3b), similar to the BNT case. Despite the application of a higher value of Δ*E*_b_ compared to that used in condition number 2, the observed unaccomplished reaction may be accounted for by the effect of the reduced number of ball collisions due to the lower value of *v*_t_ (=1955 s^−1^), which was close to half that of condition number 2. Such a sluggish reaction was more evident for condition number 4 with a higher value of Δ*E*_b_ (=1209 mJ/hit) and lower *v*_t_ values (=1105 s^−1^) (Figure S3c).

**Figure 3:**
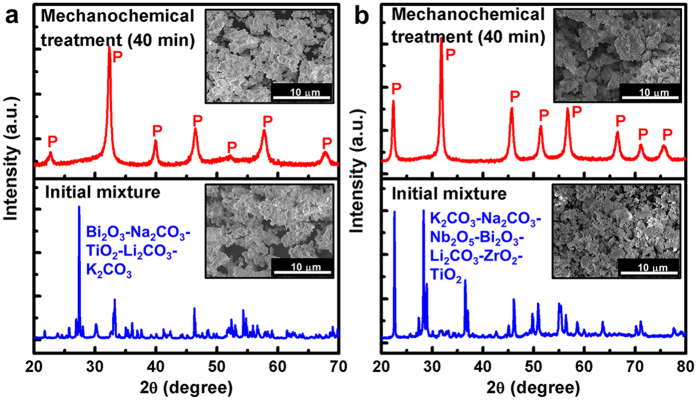
XRD patterns of the stoichiometric powder mixtures before and after a mechanochemical treatment for 40 min under condition number 2. (**a**) Bi_2_O_3_-Na_2_CO_3_-TiO_2_-Li_2_CO_3_-K_2_CO_3_. (**b**) K_2_CO_3_-Na_2_CO_3_-Nb_2_O_5_-Bi_2_O_3_-Li_2_CO_3_-ZrO_2_-TiO_2_. The insets are SEM images of each powder mixture.

Additionally, we investigated the possibility of synthesizing more complex compositions, specifically, 0.76BNT-0.04BLT-0.2BKT and 0.955KNN-0.03BNKLZ-0.015BNT, under an identical milling condition (40 min under condition number 2). Figure 3a and b show that the relatively well-defined XRD peaks precisely correspond to a perovskite structure without any second phases in both compositions. The crystallite sizes of the perovskite phase were *ca*. 16.4 nm for the BNT-BLT-BKT and *ca*. 17.6 nm for the KNN-BNKLZ-BNT, similar to those of the unmodified powders. These results confirmed that the rapid formation of nano-crystalline perovskite oxides by a high-rate mechanochemical reaction was highly reproducible, even if many reactants were involved. Promisingly, the minor influence of the number of starting reactants and the induced complexity of the composition on the formation of the perovskite structure offer more freedom in the choice of compositions in a multi-component oxide study.

Regarding contamination from the milling medium during milling, no trace of WC was visible in the XRD patterns of any of the powders prepared in this work (condition numbers 1, 2, and 3). An additional ICP analysis revealed that the amounts of tungsten (W) and cobalt (Co) as possible contaminants were at an acceptable level of less than 100 ppm and 20 ppm, respectively. However, prolonged milling with an excessive value of Δ*E*_b_ as in the case of condition number 4 induced contamination from the milling medium (Figure S3c).

The observed drastic structural change, leading to the rapid formation of single-phase perovskite oxides, was obviously a result of a high mechanochemical reaction rate among the reactants of a type not normally observed in previous mechanochemical studies or under conventional milling conditions. Regardless of the number and nature of the reactant powders, such a high-rate solid-solution reaction process was realized by enhancing the key kinetic parameters of Δ*E*_b_ and *v*_t_. The underlying mechanisms could be understood on the basis of defect-enhanced solid-state reaction processes in which the reactant particles undergo a significant size reduction via the fracturing and generation of sub-grain structures, typically leading to a nano-crystalline or amorphous structure and hence the enhanced degree of solid-state diffusivity and chemical reactivity.

The XRD results for the different milling conditions always indicated the presence of a nano-crystalline or amorphous state, generally referred to as “mechanochemically activated” in other studies^3^. This state usually appeared before the first appearance of the crystalline perovskite product. This trend was sensitive to the applied Δ*E*_b_, determining the mechanochemical reaction pathway. No chemical reaction for the formation of the perovskite product could commence below a certain minimum value of Δ*E*_b_, as shown by the syntheses of BNT and KNN under condition numbers 5 and 6 using low-density YSZ balls (Figure S4a and b). Moreover, at these energy levels, the perovskite product could be governed neither by *v*_t_ nor by *E*_cum_; the values of *v*_t_ and *E*_cum_ utilized for condition number 5 showing no formation of the perovskite BNT and KNN were sufficiently high at *v*_t_ = 72250 s^−1^ and *E*_cum_ = 2800 kJ/g. It is therefore reasonable to assume that a critical ball-impact energy *E*_c_, above or below which either a nano-crystalline/amorphous or a perovskite product is formed, exists for reactions (1) and (2), and this critical energy is correlated with an intrinsic activation energy barrier possessed by each reaction. Although the precise determination of *E*_c_ was challenging due to the limited data available from the present sets of experiments, the minimum energy requirement (*i.e*., *E*_c_) to trigger the reactions was clearly material-dependent, that is, higher for KNN (46.4–115 mJ/hit) than for BNT (10.8–46.4 mJ/hit) based on the XRD results. We also note that the observed energy levels were within a similar range of existing ball-impact energy levels applied in previous mechanochemical studies of several oxide systems^22^,^33^,^41^,^42^. On the other hand, once the applied Δ*E*_b_ exceeds *E*_c_ and hence the balls collide with sufficiently high kinetic energy for the reaction to be successful, the mechanochemical reaction can then be promoted by enhancing *v*_t_. This situation was evidenced by comparing the case of condition number 2 with those of condition numbers 3 and 4 with higher Δ*E*_b_ and lower *v*_t_ values, demonstrating the presence of certain unreacted oxide reactants (*e.g*., TiO_2_, Nb_2_O_5_). By clarifying the individual roles of Δ*E*_b_ and *v*_t_ during the mechanochemical reaction, our findings also contradict earlier reports by Abdellaoui *et al*.^27^,^43^, who reported that the cumulative kinetic energy *E*_cum_ or shock power, defined as the product of Δ*E*_b_ and *v*_t_, is the only factor responsible for the formation of the end product.

**Figure 4:**
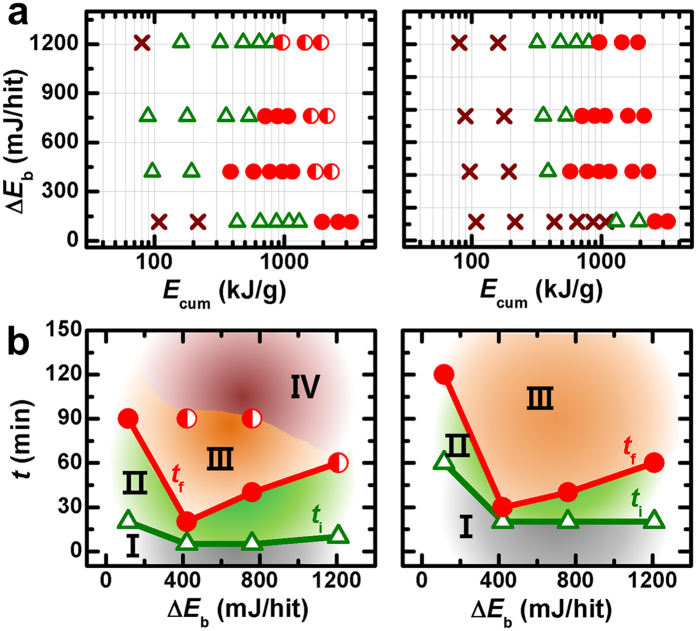
(**a**) Energy maps (Δ*E*_b_ *vs. E*_cum_) for the mechanochemical formation of perovskite BNT (left) and KNN (right). The presence of the perovskite phase was assessed from the XRD patterns with different milling conditions: not formed (×), perovskite with an unreacted phase (∆), pure perovskite (●), perovskite with a further reacted phase (◐). (**b)** Plots for *t* vs. Δ*E*_b_ showing the formation regions of perovskite BNT (left) and KNN (right) (region I: no perovskite, region II: perovskite + reactants, region III: perovskite, region IV perovskite + a further reacted phase). Here, the *t*_i_ and *t*_f_ are the milling times required for the initial appearance and the complete formation of the perovskite product, respectively.

In Fig. 4a, the energy map, *i.e*., Δ*E*_b_ (equation 3) *vs. E*_cum_ (equation 5), was constructed by assessing the presence of perovskite BNT and KNN phases based on the XRD patterns with different milling conditions. The resulting maps fully defined the energy regions required for both the initiation and, more importantly, completion of the perovskite product of each oxide system: none of the studies showed such complete maps, only providing evidence of the initiation of the perovskite product for several oxide systems owing to limited kinetic energy levels^33^. From these maps, the milling times required for the initiation (*t*_i_) and completion (*t*_f_) of each perovskite product were determined with different values of Δ*E*_b_ (Fig. 4b). Both *t*_i_ and *t*_f_ varied significantly with Δ*E*_b_, revealing different reaction thresholds and ends for each oxide system. The shortest *t*_i_ (*ca*. 5 min for BNT and *ca*. 20 min for KNN) and *t*_f_ (*ca*. 20 min for BNT and *ca*. 30 min for KNN) observed for condition number 2 suggested that the reaction rate was the highest. However, the reaction rate decreased for conditions 1, 3 and 4 with increases in *t*_i_ and *t*_f_. These results clearly suggest that the interplay between Δ*E*_b_ and *v*_t_ strongly affects the reaction rate, demanding the proper conditions between them.

**Figure 5:**
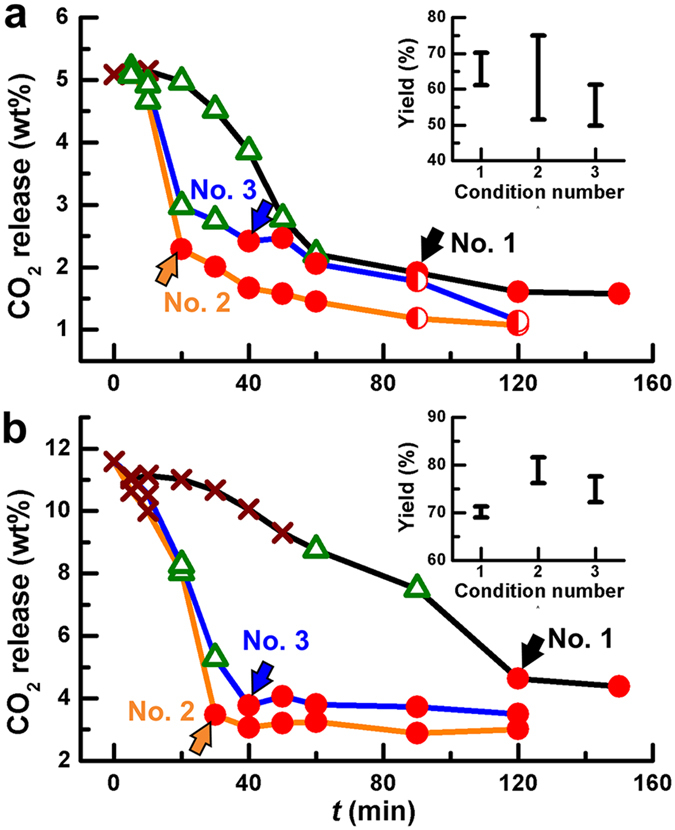
Variation of the weight loss of the milled powders owing to the release of CO_2_ as a function of the milling time *t* under different milling conditions. (**a**) The stoichiometric Bi_2_O_3_-Na_2_CO_3_-TiO_2_ powder mixture. (**b**) The stoichiometric K_2_CO_3_-Na_2_CO_3_-Nb_2_O_5_ powder mixture. The symbols showing the formation of the perovskite phase with the milling time in Fig. 4 were overlapped with each TGA curve. The arrows indicate the activation periods necessary for the dominant formation of perovskite products under each milling condition. The insets show the reaction yields taken from the perovskite periods (steady conditions) of condition numbers 1, 2 and 3.

The milling-time-dependent TGA scans provided important information about the mechanochemical reaction of the powder mixtures. Because the evolution of CO_2_ gas with a loss of weight is always indicative of the occurrence of chemical reactions (1) and (2), we were able to evaluate the degree of the completion of the reactions from the amount of CO_2_ gas released from the thermal decomposition of the carbonates remaining in the milled powder. The TGA curves (Figs 5 and S5) show a decrease in the weight loss of both powder mixtures with a longer milling time. This trend implies the occurrence of reactions (1) and (2) for the formation of the perovskite BNT and KNN. The overall weight losses of the non-milled Bi_2_O_3_-Na_2_CO_3_-TiO_2_ and K_2_CO_3_-Na_2_CO_3_-Nb_2_O_5_ mixtures amounted to *ca*. 5.09% and *ca*. 11.58%, respectively, slightly higher than those calculated when assuming complete decomposition of the alkali metal carbonates, *i.e*., *ca*. 4.94% for Bi_2_O_3_-Na_2_CO_3_-TiO_2_ and *ca*. 11.35% for K_2_CO_3_-Na_2_CO_3_-Nb_2_O_5_. The small discrepancy between the theoretical and experimental values may have originated from the evaporation of small amounts of H_2_O adsorbed by the hygroscopic reactants during the preparation processes. Notably, their respective weight losses greatly decreased to *ca*. 2.07% and *ca*. 3.07% after 40 min of milling under condition number 2.

For both of the powder mixtures (Fig. 5a and b), there was a common trend in that the carbon content gradually decreased at a certain milling time, after which there was a sudden decrease followed by no significant change, suggesting that the reaction approached a steady state^23^. The observed sudden change was clearly caused by the intense formation of perovskite BNT and KNN products from the accelerated mechanochemical reaction. Furthermore, the existence of a certain milling duration when this sudden drop occurred implied that the mechanochemical reaction began with an activation period, during which the size reduction and crystal deformation processes continually took place to create chemically active sites, eventually facilitating the chemical reactions. According to Fig. 5, the activation period of reaction (2) for the predominant formation of KNN under condition number 2 was *ca*. 30 min, which was longer than that of reaction (1) for BNT (*ca*. 20 min); for condition numbers 1 and 3 with lower and higher values of Δ*E*_b_, respectively, the activation periods became longer. All of these results were consistent with the XRD behavior (Figs 1a and 2a, S2a and S3a). Consequently, the cumulative kinetic energy *E*_cum_ necessary to overcome such activation periods, characterized by a nano-crystalline or an amorphous state based on the XRD results, was calculated and found to be *ca*. 387 kJ/g for the BNT and *ca*. 580 kJ/g for the KNN for condition number 2. The degree of the completion of the reaction, *i.e*., the reaction yield, was determined from the perovskite periods (steady levels) for each condition (insets of Fig. 5). For condition number 2, these values were *ca*. 63.3 ± 11.7% for BNT and *ca*. 78.9 ± 2.7% for KNN. The lower reaction yield observed for BNT, despite the fact that its formation was easier than that for KNN (Fig. 4), may have been related to the formation of a further reacted Bi phase. Although the Bi phase was invisible within the perovskite periods in the XRD patterns, we cannot exclude the possibility of the coexistence of a Bi phase with the perovskite BNT before its crystallization. In contrast, the KNN showed a predominant formation of perovskite products. Nevertheless, some carbonates, although undetectable by our XRD system due to their amorphous characteristics, likely did not decompose. Considering the required milling time for different conditions, the reaction efficiency (*i.e*., the ratio of the reaction yield to the milling time) was also highest for condition number 2.

Conclusively, we ascribe the present findings of a much shorter reaction time (*ca*. 30–40 min under condition number 2) necessary to stabilize a single-phase perovskite structure to the highly energetic milling conditions using the greater rotational speed of the disk (1000 rpm), which allows an enhanced reaction rate induced by much more vigorous ball collisions at more than a certain minimum level of kinetic energy. Given equations 3,4,5, the most critical milling parameter to increase both Δ*E*_b_ and *v*_t_ is known to be *W*_p_. For condition number 2, equation 5 approaches this, as shown below.


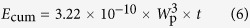


**Figure 6:**
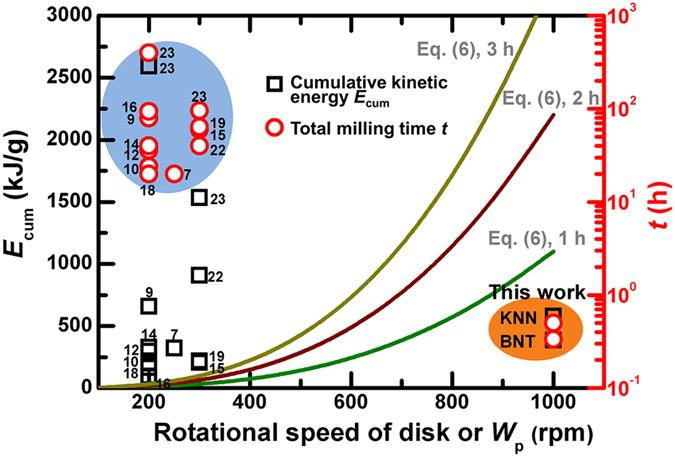
Comparison of the cumulative kinetic energy *E*_cum_ and the milling time *t* taken from the literature (Table 1) with those used in this work. The literature data were limited to those corresponding to the dominant formation of perovskite oxides.

This relationship implies that *E*_cum_ is highly sensitive to *W*_p_ with linear *W*_p_^3^ dependence; see the fitted curves of *E*_cum_ *vs. W*_p_ in Fig. 6 when *t* = 1, 2 and 3 h. In this figure, the literature data pertaining to *E*_cum_ and *t* required for the dominant formation of various perovskite oxides are also presented and compared to those used in this work. The prolonged period of milling between 20–400 h needed in many mechanochemical studies is attributed to the lower level of *W*_p_ (200–300 rpm). For instance, the milling time of 400 h required to reach an *E*_cum_ value of 2592 kJ/g at a disk speed of 200 rpm (used in ref. 23) can be dramatically reduced to less than 3 h when adopting condition number 2 as defined in this work.

**Figure 7:**
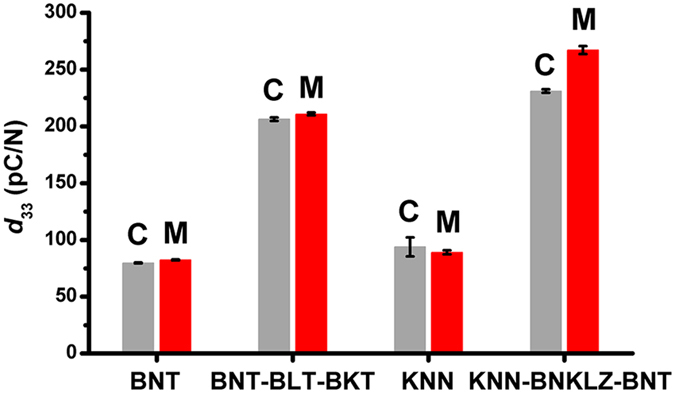
Piezoelectric coefficient *d*_33_ of ceramics sintered using powders prepared both by mechanochemical synthesis for 40 min (denoted by “M”) and conventional solid-state synthesis (denoted by “C”).

Using mechanochemically derived powders, the sintering characteristics were investigated and compared to those obtained from a conventional solid-state reaction process. All of the ceramics including the BNT, BNT-BLT-BKT, KNN, and KNN-BNKLZ-BNT types, had pure perovskite structures without any second phases (Figure S7). The absence of changes in the peak positions or intensities for the ceramics prepared by both methods indicated nearly identical structural characteristics (BNT, monoclinic; BNT-BLT-BKT, pseudo-cubic^44^; KNN, orthorhombic; KNN-BNKLZ-BNT, diphasic rhombohedral-tetragonal^36^). According to SEM backscattered electron images (BEIs) of the ceramics prepared by mechanochemical synthesis, the homogeneously distributed main elements confirmed nearly complete perovskite solid solutions, together with the formation of well-developed perovskite grains with clear edges, similar to those prepared by conventional solid-state synthesis (Figure S8). The values of the piezoelectric coefficient *d*_33_ obtained for the ceramics prepared by mechanochemical synthesis were similar to or slightly higher than those for the ceramics prepared by the conventional powder process (Fig. 7). Particularly, the modified BNT-BLT-BKT and KNN-BNKLZ-BNT ceramics prepared by mechanochemical synthesis showed excellent *d*_33_ values of *ca*. 211 ± 1.2 pC/N and *ca*. 267 ± 3.5 pC/N, respectively. These values are comparable to the best properties reported for identical material systems prepared by the conventional solid-state synthesis method (*ca*. 190 pC/N by Hiruma *et al*.^35^ and *ca*. 147–231 pC/N by Lin *et al*.^45^ for BNT-BLT-BKT and *ca*. 285 pC/N by Cheng *et al*.^36^ for KNN-BNKLZ-BNT).

In summary, we have demonstrated that complex perovskite oxides can be expeditiously synthesized from micron-sized reactant powders via a mechanochemical reaction using a highly energetic ball milling process. Only a very short milling time of *ca*. 30–40 min was required to yield nano-crystalline, single-phase perovskite BNT and KNN oxides reliably, even given their more complex compositions. This was enabled by the high mechanochemical reaction rate induced by enhancements of key kinetic parameters, in this case the ball-impact energy (Δ*E*_b_ = 421 mJ/hit) and frequency (*v*_t_ = 3825 s^−1^). From the mechanochemical reaction outcomes with different values of Δ*E*_b_ and v_t_, it was demonstrated that Δ*E*_b_ should be higher than a critical kinetic energy *E*_c_ (10.8–46.4 mJ/hit for BNT, 46.4–115 mJ/hit for KNN) to initiate the mechanochemical formation of the perovskite product. Additionally, the mechanochemical reaction could be promoted by enhancing *v*_t_, but only if the balls had sufficiently high kinetic energy for the reaction to occur. According to the TGA results, the formation of perovskite BNT and KNN required an activation period, during which the solid-state chemical reactivity of the reactant powders continued to increase, ultimately inducing the intense formation of the perovskite product. Furthermore, the formation of BNT was easier than that of KNN in terms of both the energy requirement and the activation period. The values of *E*_cum_ necessary to overcome the activation period for the formation of a dominant perovskite structure were *ca*. 387 kJ/g for BNT and *ca*. 580 kJ/g for KNN. Finally, the mechanochemically derived perovskite powders, when consolidated into bulk ceramics, showed encouraging piezoelectric properties that were not inferior to those prepared by conventional solid-state synthesis. Benefitting from the ability to skip the conventional high-temperature and time-consuming powder procedures, the high-rate mechanochemical route used here is not limited to the presently investigated oxides. It can be readily extended to other technologically important multi-component oxides, possibly irrespective of the choice of composition or the nature of the reactant (*e.g*., chemical stability).

## Additional Information

**How to cite this article:** Lee, G.-J. *et al*. Rapid and direct synthesis of complex perovskite oxides through a highly energetic planetary milling. *Sci. Rep.* **7**, 46241; doi: 10.1038/srep46241 (2017).

**Publisher's note:** Springer Nature remains neutral with regard to jurisdictional claims in published maps and institutional affiliations.

## Supplementary Material

Supplementary Information

## Figures and Tables

**Table 1:** Kinetic parameters of the high-energy planetary ball milling process for the mechanochemical synthesis of various perovskite oxides.

Material^*a*^	Mill^*b*^	Ball/*d*_b_ (mm)	Disk speed (rpm)	Δ*E*_b_ (mJ/hit)^*c*^	*v*_t_ (s^−1^)^*d*^	*t* (h)	*E*_cum_ (kJ/g)^*e*^	Product^*f*^	ref
BNT/KNN	No. 1	WC/5	1000	115	15725	2.5	3257(1303)	●/○	This work
BNT/KNN	No. 2	WC/8	1000	421	3825	2	2320(1160)	●/●	This work
BNT/KNN	No. 3	WC/10	1000	760	1955	2	2140(1070)	●/●	This work
BNT/KNN	No. 4	WC/12	1000	1209	1105	2	1924(962)	●/●	This work
BNT/KNN	No. 5	YSZ/3	1000	10.8	72250	5	2800(560)	×/×	This work
BNT/KNN	No. 6	YSZ/5	1000	46.4	15725	2.5	1312.5(525)	○/×	This work
PT	P5	WC/20	250	671	200	20	322(16.1)	○	[7]
PZ	P5	WC/20	200	429	160	24	198(8.2)	∆	[8]
PZT	P5	WC/20	200	429	160	80	659(8.2)	○	[9]
PZT	P5	WC/20	200	429	160	24	198(8.2)	○	[10]
PLZT	P5	WC/20	200	429	160	36	297(8.2)	○	[12]
PLZT	PM400	WC/10	200	43	1500	10	77(7.7)	∆	[13]
PMN	P5	WC/20	200	429	160	40	329(8.2)	○	[14]
PMN	PM400	WC/20	300	560	340	60	206(3.4)	○	[15]
PMN	PM400	WC/20	200	250	230	94	97(1.0)	○	[16]
PMN-PT	P5	WC/20	200	429	160	20	165(8.2)	○	[18]
PMN-PT	PM400	WC/20	300	560	340	64	219(3.4)	○	[19]
BT	PM400	WC/10	200	43	1500	10	77(7.7)	×	[20]
NN	PM400	YSZ/10	300	35	900	40	907(22.7)	○	[22]
NN	PM400	YSZ/10	200	15	600	400	2592(6.5)	●	[23]
NN	P4	WC/15	300	370	360	96	1534(16.0)	●	[23]
KN	PM400	YSZ/10	300	35	900	40	907(22.7)	×	[22]
KN	P4	WC/15	270	300	320	350	12096(34.6)	∆	[24]
KNN	PM400	YSZ/10	300	35	900	10	227(22.7)	×	[22]

^a^BNT: (Bi, Na)TiO_3_, KNN: (K, Na)NbO_3_, PT: PbTiO_3_, PZ: PbZrO_3_, PZT: Pb(Zr, Ti)O_3_, PLZT: (Pb, La)(Zr, Ti)O_3_, PMN: Pb(Mg, Nb)O_3_, PMN-PT: Pb(Mg, Nb)O_3_-PbTiO_3_, BT: BaTiO_3_, NN: NaNbO_3_, KN: KNbO_3_.

^b^All mills including that used in this work were of the planetary ball-mill type: P4 (Fritsch Pulverisette 4), P5 (Fritsch Pulverisette 5), and PM400 (Retsch PM400).

^c^Ball-impact energy^32^:
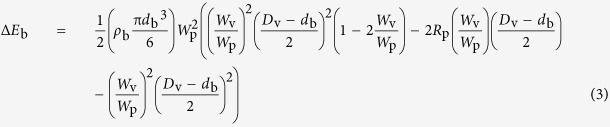

^d^Ball-impact frequency^32^:
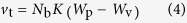

^e^Cumulative kinetic energy^32^:
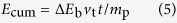

Here, *ρ*_b_ is the density of the balls, *d*_b_ is the diameter of the balls, *W*_p_ is the rotational angular speed of the supporting disk, *W*_v_ is the rotational angular speed of the vial, *D*_v_ is the diameter of the vial, *R*_p_ is the distance between the rotational axes, *N*_b_ is the number of balls, *K* is a constant, *t* is the milling time and *m*_p_ is the powder weight. The constant *K* was 1.5 cited from ref. 46. For other studies, the values of *E*_cum_ calculated assuming 1 h of milling are given in the parentheses for comparison with those of this work.

^*f*^The mechanochemical formation of the perovskite product was assessed based on the reported XRD patterns: ● (phase-pure), ○ (dominant but with contamination or an unreacted phase), ∆ (partly formed), × (not formed).
