# Supplementary material for: Rapid and direct synthesis of complex perovskite oxides through a highly energetic planetary milling
Source: Sci Rep. 2017 Apr 7;7:46241. doi: 10.1038/srep46241 (PMC5384223; doi:10.1038/srep46241)
Supplement: Supplementary Information [file srep46241-s1.doc]

**Supplementary Information**

Rapid and direct synthesis of complex perovskite oxides through a highly energetic planetary milling

Gyoung-Ja Lee^1^, Eun-Kwang Park^1^, Sun-A Yang^1^, Jin-Ju Park^1,*^, Sang-Don Bu^2^ & Min-Ku Lee^1,*^

1 Nuclear Materials Development Division, Korea Atomic Energy Research Institute, 989-111 Daedeok-daero, Yuseong-gu, Daejeon 34057, Republic of Korea

2 Department of Physics and Research Institute of Physics and Chemistry, Chonbuk National University, Jeonju 54896, Republic of Korea

* corresponding author: leeminku@kaeri.re.kr


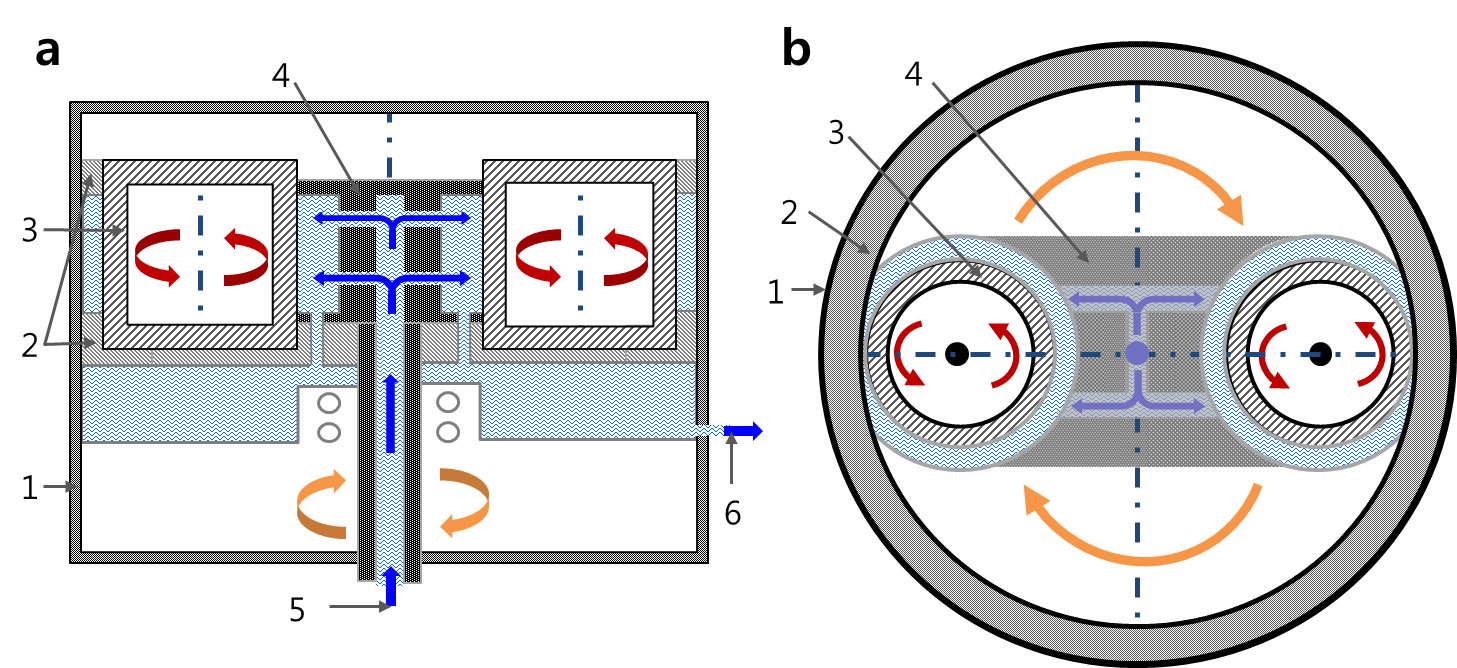


**Figure S1.** Schematic diagrams of a) front and b) top sides of the high energy milling system: 1 (housing), 2 (guides), 3 (vial), 4 (supporting disk), 5 (cooling water inlet), 6 (cooling water outlet).

Figure S1 shows schematic diagram of high energy milling system. In order to provide a much higher milling energy, two main design concepts were considered: mechanical balance stabilization by employing the vial-embedded supporting disk and efficient water cooling system for the prevention of temperature increase inside a vial.

**Figure S2.** XRD patterns of the stoichiometric Bi_2_O_3_-Na_2_CO_3_-TiO_2_ powder mixture mechanochemically treated as a function of the milling time. a) Condition number 1 (Δ*E*_b_ = 115 mJ/hit, *v*_t_ = 15725 s^-1^). b) Condition number 3 (Δ*E*_b_ = 760 mJ/hit, *v*_t_ = 1955 s^-1^). c) Condition number 4 (Δ*E*_b_ = 1209 mJ/hit, *v*_t_ = 1105 s^-1^).

Figure S2 shows the XRD formation behavior of the stoichiometric Bi_2_O_3_-Na_2_CO_3_-TiO_2_ powder mixture after mechanochemical treatments as a function of the milling time under condition numbers 1, 3 and 4. For condition number 1 (Figure S2a), the TiO_2_ and amorphous structure were detected until 10 min. After 20 min, the perovskite BNT began to appear with TiO_2_, an amorphous phase and the intermediate Bi_4_Ti_3_O_12_. After 40 min, the Bi_4_Ti_3_O_12_ phase disappeared and only the perovskite and TiO_2_ remained. Subsequently, the perovskite BNT was dominant but with the presence of a small amount of TiO_2_ until 150 min. For condition number 3 (Figure S2b), only 5 min of milling produced the perovskite BNT together with TiO_2_, an amorphous phase and Bi_4_Ti_3_O_12_. After 20 min, the perovskite BNT was dominant; however, the Bi phase began to appear at 120 min of milling, possibly resulting from a further decomposition of the perovskite BNT. For condition number 4 (Figure S2c), this trend became prominent, showing the formation of the Bi phase via the formation of perovskite BNT at 60 min, after which the intensity of the Bi phase increased with an increase in the milling time. The transition of the perovskite BNT into Bi phase appeared to be rapid under this condition, scarcely showing a perovskite-pure region. After 60 min, WC was also detected as a contaminant from the milling medium, possibly due to the effect of the higher ball-impact energy (Δ*E*_b_ = 1209 mJ/hit).

**Figure S3.** XRD patterns of the stoichiometric K_2_CO_3_-Na_2_CO_3_-Nb_2_O_5_ powder mixture mechanochemically treated as a function of the milling time. a) Condition number 1 (Δ*E*_b_ = 115 mJ/hit, *v*_t_ = 15725 s^-1^). b) Condition number 3 (Δ*E*_b_ = 760 mJ/hit, *v*_t_ = 1955 s^-1^). c) Condition number 4 (Δ*E*_b_ = 1209 mJ/hit, *v*_t_ = 1105 s^-1^).

Figure S3 shows the XRD formation behavior of the stoichiometric K_2_CO_3_-Na_2_CO_3_-Nb_2_O_5_ powder mixture after mechanochemical treatments as a function of the milling time under condition numbers 1, 3 and 4. For condition number 1 (Figure S3a), gradual decreases in the stable Nb_2_O_5_ peaks were observed with an increase in the milling time to 90 min and at the same time the amorphous structure became severe. The perovskite KNN phase appeared after 60 min, finally becoming dominant after 120 min. For condition number 3 (Figure S3b), the phase evolution trend was similar, but more rapid. The milling time necessary for the dominant formation of the perovskite structure was longer than that for condition number 2, which was ca. 40 min. For condition number 4 (Figure S3c), the Nb_2_O_5_ remained unreacted until 50 min, after which the perovskite structure was dominant. Despite the higher Δ*E*_b_, a sluggish reaction was evident compared to that in condition number 3, which may have been due to the reduced number of ball collisions due to the lower value of *v*_t_ (= 1105 s^-1^). Unlike the case of BNT, no further reacted phase was observed.

**Figure S4.** XRD patterns of the milled powders mechanochemically treated using YSZ balls as a function of the milling time under condition numbers 5 (Δ*E*_b_ = 10.8 mJ/hit, *v*_t_ = 72250 s^-1^) and 6 (Δ*E*_b_ = 46.4 mJ/hit, *v*_t_ = 15725 s^-1^). a) The stoichiometric Bi_2_O_3_-Na_2_CO_3_-TiO_2_ powder mixture. b) The stoichiometric K_2_CO_3_-Na_2_CO_3_-Nb_2_O_5_ powder mixture.

Figures S4a and b show the XRD formation behaviors of the milled powders mechanochemically treated at lower levels of Δ*E*_b_ using low-density YSZ balls (6.08 g/cm^3^ in density *ρ*_b_). For the stoichiometric Bi_2_O_3_-Na_2_CO_3_-TiO_2_ powder mixture, condition number 6 (Δ*E*_b_ = 46.4 mJ/hit, *v*_t_ = 15725 s^-1^) induced the dominant formation of the perovskite structure after 150 min of milling. In contrast, no formation of the perovskite BNT occurred under condition number 5 (Δ*E*_b_ = 10.8 mJ/hit, *v*_t_ = 72250 s^-1^); instead, the amorphous structure was visible until 300 min. Despite the presence of a higher *E*_cum_ value (= 2800 kJ/g) for condition number 5 as compared to that for condition number 6 (*E*_cum_ =1312.5 kJ/g), the absence of the formation of the perovskite product clearly indicated that the reaction for the perovskite product was not governed by *E*_cum_. These results also indicated that the level of *E*_c_ must be between 10.8 and 46.4 mJ/hit. It was also noted that this energy level was within a similar range of the energy levels applied in previous studies, in which none of the cases showed the formation of the perovskite product with several oxide systems such as SrTiO_3_,^1^ Sr_2_TiO_4_,^1^ BaTiO_3_,^2^ LiMn_2_O_4_,^3^ LiMnO_2_,^3^ Li_2_MnO_3_,^3^ and BaFe_12_O_19_^4^. On the other hand, for the stoichiometric K_2_CO_3_-Na_2_CO_3_-Nb_2_O_5_ powder mixture, conditions numbers 5 and 6 virtually failed to produce the perovskite KNN, only resulting in an amorphous structure together with the presence of a crystalline reactant phase. Moreover, for condition number 5, nearly identical XRD patterns were observed when the milling time was increased from 150 min to 300 min, indicating no change in the structural characteristics. For condition number 6 with a higher value of Δ*E*_b_, the only notable change was the slightly reduced intensity of the reactant peaks. This clearly indicated that the values of the applied value of Δ*E*_b_ were insufficient to cause reaction (2) and that the *E*_c_ value necessary for the formation of perovskite KNN should be higher than that for BNT. Considering the effects of *E*_cum_ and *v*_t_, neither *E*_cum_ nor *v*_t_ governed the related mechanochemical reaction for the perovskite BNT and KNN. Instead, Δ*E*_b_ was the critical parameter determining the reaction pathway.

**Figure S5.** TG curves of the stoichiometric Bi_2_O_3_-Na_2_CO_3_-TiO_2_ powder mixture as a function of the milling time. a) Condition number 1 (Δ*E*_b_ = 115 mJ/hit, *v*_t_ = 15725 s^-1^). b) Condition number 2 (Δ*E*_b_ = 421 mJ/hit, *v*_t_ = 3825 s^-1^). c) Condition number 3 (Δ*E*_b_ = 760 mJ/hit, *v*_t_ = 1955 s^-1^). d) Condition number 4 (Δ*E*_b_ = 1209 mJ/hit, *v*_t_ = 1105 s^-1^).

**Figure S6.** TG curves of the stoichiometric K_2_CO_3_-Na_2_CO_3_-Nb_2_O_5_ powder mixture as a function of the milling time. a) Condition number 1 (Δ*E*_b_ = 115 mJ/hit, *v*_t_ = 15725 s^-1^). b) Condition number 2 (Δ*E*_b_ = 421 mJ/hit, *v*_t_ = 3825 s^-1^). c) Condition number 3 (Δ*E*_b_ = 760 mJ/hit, *v*_t_ = 1955 s^-1^). d) Condition number 4 (Δ*E*_b_ = 1209 mJ/hit, *v*_t_ = 1105 s^-1^).

Figures S5 and 6 show the TGA curves of the stoichiometric Bi_2_O_3_-Na_2_CO_3_-TiO_2_ and K_2_CO_3_-Na_2_CO_3_-Nb_2_O_5_ powder mixtures, respectively, as a function of the milling time at different milling conditions. From all of the measured TGA curves, the weight loss by CO_2_ release was determined. These data are presented in Figure 5. The increase in weight loss with the milling time was clear, indicating not only the occurrence of reactions (1) and (2) but also increased yields of the perovskite BNT and KNN products owing to the completion of the related reactions. The existence of a certain temperature approaching a steady level meant the completion of the reactions for the formation of the perovskite BNT and KNN. It was noted that this temperature decreased as the milling time increased, confirming the effect of the mechanochemical activation of the powders.

**Figure S7.** XRD patterns of the ceramics sintered using powders prepared both by mechanochemical synthesis for 40 min (denoted by “M”) and by conventional solid-state synthesis (denoted by “C”).

Figure S7 shows XRD patterns of the sintered BNT, BNT-BLT-BKT, KNN, and KNN-BNKLZ-BNT ceramics prepared both by mechanochemical synthesis for 40 min (denoted by “M”) and by conventional solid-state synthesis (denoted by “C”). All of the ceramics had a pure perovskite structure without any second phases. The absence of changes in the peak positions or intensities for the ceramics prepared by both methods indicated nearly identical structural characteristics. The pure BNT ceramic had a monoclinic structure and the BNT-BLT-BKT ceramic had a pseudo-cubic structure with no obvious splitting of the (111) and (200) peaks, which is a characteristic of relaxor materials.^5^ The KNN ceramic had an orthorhombic structure, while the KNN-BNKLZ-BNT ceramic exhibited a diphasic rhombohedral-tetragonal structure due to the formation of a polymorphic phase boundary.^6^

**Figure S8.** SEM morphologies of the BNT, BNT-BLT-BKT, KNN and KNN-BNKLZ-BNT ceramics sintered using prepared powders both by mechanochemical synthesis for 40 min (denoted by “M”) and by conventional solid-state synthesis (denoted by “C”). The right sides of each image show the grain size distribution and average grain size obtained from the image analysis.

Figure S8 shows SEM backscattered electron images (BEIs) which display an elemental distribution of high and low atomic numbers, along with the grain size distribution obtained from the image analysis. The SEM BEIs showed that all of the main elements were homogeneously distributed in each ceramic, indicating nearly complete perovskite solid solutions. The ceramics prepared by mechanochemical synthesis had a grain structure similar to those prepared by conventional solid-state synthesis, showing well-developed perovskite grains with clear edges. According to an image analysis, the ceramics prepared by mechanochemical synthesis appeared to have smaller grain sizes than those of the ceramics prepared by the conventional powder process. The observed smaller grain sizes may have been due to the rougher interface of the grains typically formed during the milling process compared to those of conventional calcined powders prior to sintering; grain growth with a rough interface is slower than that with a well-faceted interface, most likely due to diffusion-controlled Ostwald ripening.^7^ The decreased grain size of the chemically modified ceramics was clear; for example, the grain size of the BNT decreased from 16.2 to 1.3 μm with the addition of 0.04BLT-0.2BKT. It has been reported that an increased K^+^ ion concentration suppresses the grain-growth rate of BNT^8^ and that the grain growth of KNN can be inhibited by the addition of Bi^3+^ ions.^6^

**REFERENCES**

1. V. Berbenni, A. Marini and G. Bruni, *J. Alloy Compd.* **329**, 230–238 (2001).
2. V. Berbenni, A. Marini and G. Bruni, *Thermochim. Acta* **374**, 151–158 (2001).
3. V. Berbenni and A. Marini, *J. Anal. Appl. Pyrolysis* **70**, 437–456 (2003).
4. V. Berbenni, A. Marini, N. J. Welham, P. Galinetto and M. C. Mozzati, *J. Eur. Ceram. Soc.* **23**, 179-187 (2003).
5. H. S. Han, W. Jo, J. Rӧdel, I. K. Hong, W. P. Tai and J. S. Lee, *J. Phys. Condens. Matter* **24**, 365901 (2012).
6. X. Cheng, J. Wu, X. Wang, B. Zhang, J. Zhu, D. Xiao, X. Wang, X. Lou and W. Liang, *J. Appl. Phys.* **114**, 124107 (2013).
7. S. Y. Chung, D. Y. Yoon and S. J. L. Kang, *Acta Mater.* **50**, 3361–3371 (2002).
8. E. Fukuchi and T. Kimura, *J. Am. Ceram. Soc.* **85**, 1461–1466 (2002).
